# Elastic Properties of Open Cell Metallic Foams—Modeling of Pore Size Variation Effect

**DOI:** 10.3390/ma15196818

**Published:** 2022-09-30

**Authors:** Karol Ćwieka, Jakub Skibiński

**Affiliations:** 1 Faculty of Chemical and Process Engineering, Warsaw University of Technology, Ludwika Waryńskiego 1, 00-645 Warsaw, Poland; 2 Institute of Heat Engineering, Faculty of Power and Aeronautical Engineering, Warsaw University of Technology, 21/25 Nowowiejska Street, 00-665 Warsaw, Poland

**Keywords:** elastic behavior, foams, aluminum, compression test, finite element method

## Abstract

Elastic properties of open-cell metallic foams are investigated in correlation with relative density and pore size variation. A variety of foam architectures, with open porosity above 70% (relative density below 0.30) and various pore size distributions, were modeled using Laguerre–Voronoi tessellations (LVT). The coefficient of pore volume variation, CV(V), was introduced to quantify the uniformity of designed structures and ranged between 0.5 to 2.1. Elastic behavior of the modeled foams to uniaxial compression along three orthogonal directions was analyzed using the finite element (FE) method. It is shown that Young’s modulus and Poisson’s ratio of open-cell metals is not solely a function of relative density (porosity) but the pore size variation as well. For similar porosity (approx. 74–98%), Young’s modulus and Poisson’s ratio may be reduced by approx. 25–30% and 10–25%, respectively, when CV(V) increases from 0.5 to 2.1. Furthermore, the incorporation of a relationship between Young’s modulus and the coefficient of pore volume variation to the Gibson–Ashby model is proposed.

## 1. Introduction

Open-cell metallic materials exhibit interesting conjunction of structural and functional properties. Because of porous, lightweight structure (reduced relative density), their mechanical (specific strength, i.e., strength related to density) [1,2], thermal (conductivity) [3,4], or flow (permeability, pressure drop) [5,6] properties may be tuned by the relative density and structural homogeneity. To date, a long list of applications of cellular materials has been extensively reported. Open- and closed-cell metals are widely applied as lightweight structural components [7,8,9], filters and separators [8,10], mechanical energy and sound absorbers [7,8,11,12,13], heat exchangers and insulators [7,8,14], catalysts [8,15,16], flame arresters [8,17,18], and pneumatic silencers [8]. In particular, cellular materials are encountered in biomedical applications, e.g., scaffolds for tissue engineering and bone healing [19,20].

Numerous theoretical studies have reported endeavors to scrutinize the influence of structural and geometrical features on the mechanical properties of open-cell metals. In general, their mechanical properties are strongly governed by volume fraction and material properties of the solid skeleton [2,3]. Liu and Antoniou stated that macroscopic properties of foams are explicitly dependent on the structure, only through the relative density in low-density foams, namely with relative density < 0.1. Micromechanical investigations, however, also emphasize geometrical relations between elements of the microstructure, such as struts thickness and length, size of junctions, or mass agglomeration at junctions [4,5]. Such relations may be investigated via dimensional analysis, which is an approach based on the selection of representative and parametrized patterns (unit cells), which gives an idealized representation of the architecture of cellular material when multiplied in space as a periodic alignment. The resulting model, as opposed to real open-cell foams, possesses a high level of geometrical regularity in shape and dimensions but makes it possible to estimate whether and how this relevant dimension impacts the final properties of the structure [4,6,7]. Since real foams are inherently nonuniform and random structures, it is difficult to unequivocally describe their overall geometrical characteristics and derive explicit equations describing their properties. Accurate predictions demand more representative models of geometry, resembling the complex structure of real foams. A complete understanding of the relationship between structural characteristics and mechanical properties of metallic foam structure is essential in terms of materials design for specific structural applications.

It has been broadly confirmed that the more uniform the size distribution of microstructural elements in open-cell or bulk metals is, the more optimal the mechanical properties. Presently, manufacturers dedicate their efforts mainly to obtaining open-cell metallic foams with a narrow distribution of pore size, whereas structural inhomogeneity of open porous metallic materials brings about a distinctive impact on their functional properties. In our previous publication [5], we reported how the pressure drop is affected by the pore size variation during the flow of fluid through a porous medium. Several other papers [21,22,23] presented numerical simulations of elastic properties of metallic foams with nonuniform pore size, but neither quantified the distribution nor provided a description of any relationship between them. The important aspect of variation of the pore shape was the case analyzed in [24]. Authors of [16,25,26,27,28,29] adopted geometrically regular numerical models to analyze the elastic response of open-cell structures; thus, structural nonuniformity itself was neglected.

The influence of pore size variation on elastic properties was analyzed by Redenbach et al. [30] for closed-cell aluminum foams, showing that effective Young’s modulus and Poisson’s ratio decrease with increasing variation in the cell size. Similar conclusions were derived by Zhu et al. [31], who scrutinized the cell irregularity effect in polymer open-cell foams at low compressive strains in low-density limit using stochastic models containing 27 cells generated by the Voronoi procedure. The parameter describing the foam regularity provides information about the spatial distribution of randomly sized pores but not directly about the distribution of pore size, cf. [32].

The general formulation of scaling law for Young’s modulus of cellular materials has been derived by Gibson and Ashby [33] in the form of following the equation:

(1) $$\frac{E_{eff}}{E_{S}}=C{\left(\frac{\rho }{\rho_{S}}\right)}^{n}$$

where E_eff_ is the effective Young’s modulus of foam structure normalized with Young’s modulus of bulk E_S_, ρ/ρ_S_ is the relative density of the foam, and C and n are constants dependent on the structure. For metallic open-cell foams, the exponent n is usually assumed to be equal to 2, which indicates a quadratic relationship. Its value is mainly related to the deformation mechanism. When the struts are not aligned in the foam structure, bending at their intersection points (strut joints) or bending of the struts becomes dominant [33,34,35], and quadratic dependence of Young’s modulus on the relative density occurs. By contrast, the value of the C constant is in the range between 0.1 and 4.0 [36].

In the present study, we turn our interest to the effect of pore size variations on the elastic properties of open-cell metallic foams. A set of representative models of aluminum foams have been developed with porosity ranging from 74% to 98% and coefficient of variation of pore volume CV(V), over the range of 0.5 to 2.1. The model structures designed using the LVT algorithm were subsequently analyzed employing FEM simulations under uniaxial compression along three orthogonal directions. Based on the results of FEM analyses, the effective Young’s modulus *E_eff_* and Poisson’s ratio ν_eff_ were determined as a function of relative density and pore size variation. Computational results were fitted to the Gibson–Ashby model to provide an extended definition of the fitting parameters C and n as a function of CV(V).

## 2. Materials and Methods

**Figure 1:**
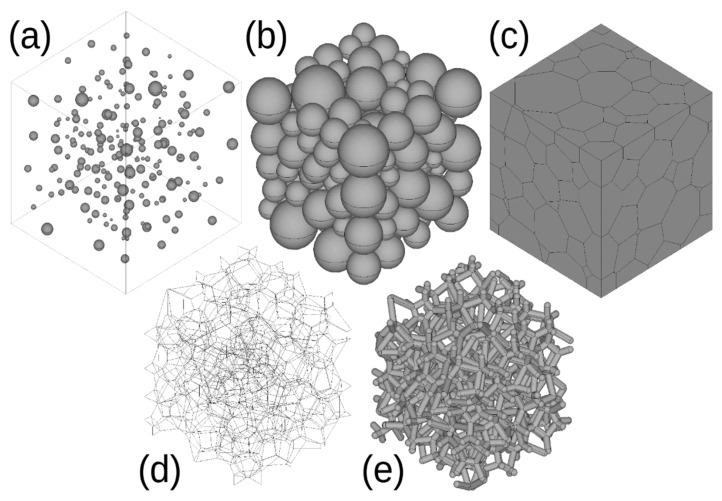
Schematic explanation of the procedure used for the generation of open-cell foams: (**a**) set of spheres with predetermined sphere size distribution in the bounding box, (**b**) spheres after aggregation, (**c**) cellular polycrystalline structure obtained using Laguerre–Voronoi tessellations, (**d**) edge model of cellular structure, (**e**) open-cell structure with cylindrical struts, and (**e**) meshed model.

Open-cell metal structures were generated using Laguerre–Voronoi tessellations (hereinafter LVT) [37,38,39]. In the first step, a set of spheres with predefined mean volume and volume variation was generated. Initially, spheres were randomly distributed in a cubic bounding box to make them fully independent with respect to their positions and provided no overlap (see Figure 1a). The bounding box was subsequently reduced in size to increase the density of spheres via their displacements towards the nearest neighbors. The termination occurred when the farther reduction was not possible (still providing no overlap between the spheres), and the fixed size of the bounding box was reached (Figure 1b).

Subsequent to the sphere-packing stage, the Laguerre–Voronoi tessellations were applied to obtain cellular structures. The cell faces were created by the intersection of the volume by planes positioned in the proportional distance between two neighboring spheres in the way that the intersecting plane between two spheres is not located at an equal distance from the centers of the spheres but in the middle of the distance between (nonoverlapping) spheres. That enables introducing the cells’ size variation to the tessellated polycrystalline structure. As a result, a model of a polycrystalline structure was generated with polygonal tessellated grains. To obtain a triple-edge open-cell structure, all grains were removed (for more details, see [38,39,40]). In the final step, triple edges were expanded into cylinders of a predetermined radius. Additionally, spheres having the same diameters as the struts were generated at fourfold points to assure dimensional conformity along the edges. All the above-listed operations were coded using the APDL language script implemented in the ANSYS software. A more detailed description of the procedure used can be found in [5,40,41].

**Figure 2:**
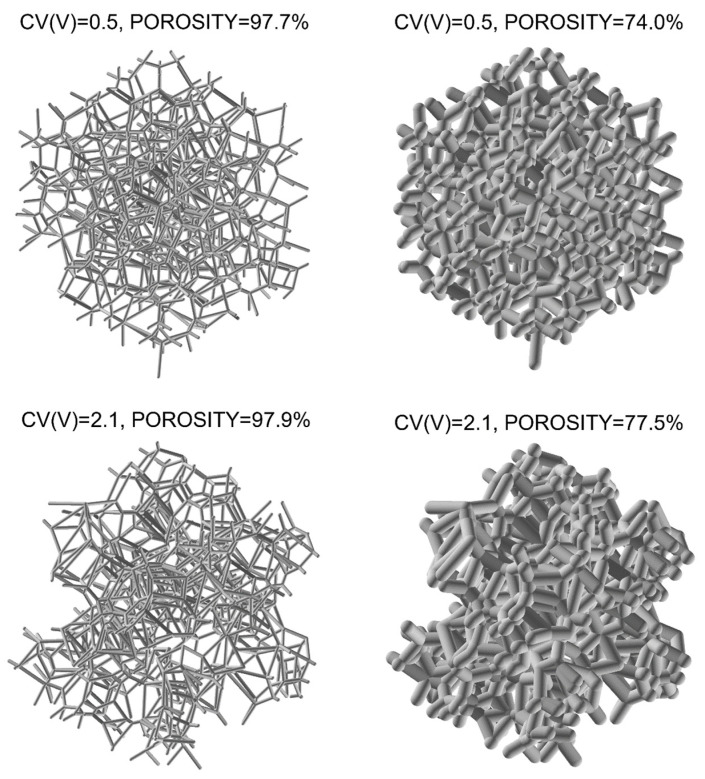
Examples of designed LVT open-cell foams with various CV(V).

The procedure described above led to the design of a set of 15 structures differing in the coefficient of pore volume variation CV(V). Values of CV(V) assumed as the ratio of the standard deviation to the average volume of the pores ranged from 0.5 to 2.1. The structure with a CV(V) of 0.5 featured the highest uniformity of pore size distribution, while the CV(V) of 2.1 had the highest diversity (see Figure 2). In order to study the influence of porosity (relative density), the four values (0.5, 1.0, 1.5, and 2.0 mm) of strut diameter were analyzed, bringing about 60 structures with the lowest porosity of approx. 74% and the highest approx. 98%. Each structure was derived from 200 spheres, and in accordance with the literature data [21,31,32,35], the number of pores was sufficient to generate representative models for the analysis of the properties of the designed structures. In particular, the results of sensitivity analyses regarding the number of cells in irregular foam structures show that calculated mean values of elastic properties are almost equal for the number of cells ranging from 27 to 512 [31,32].

**Figure 3:**
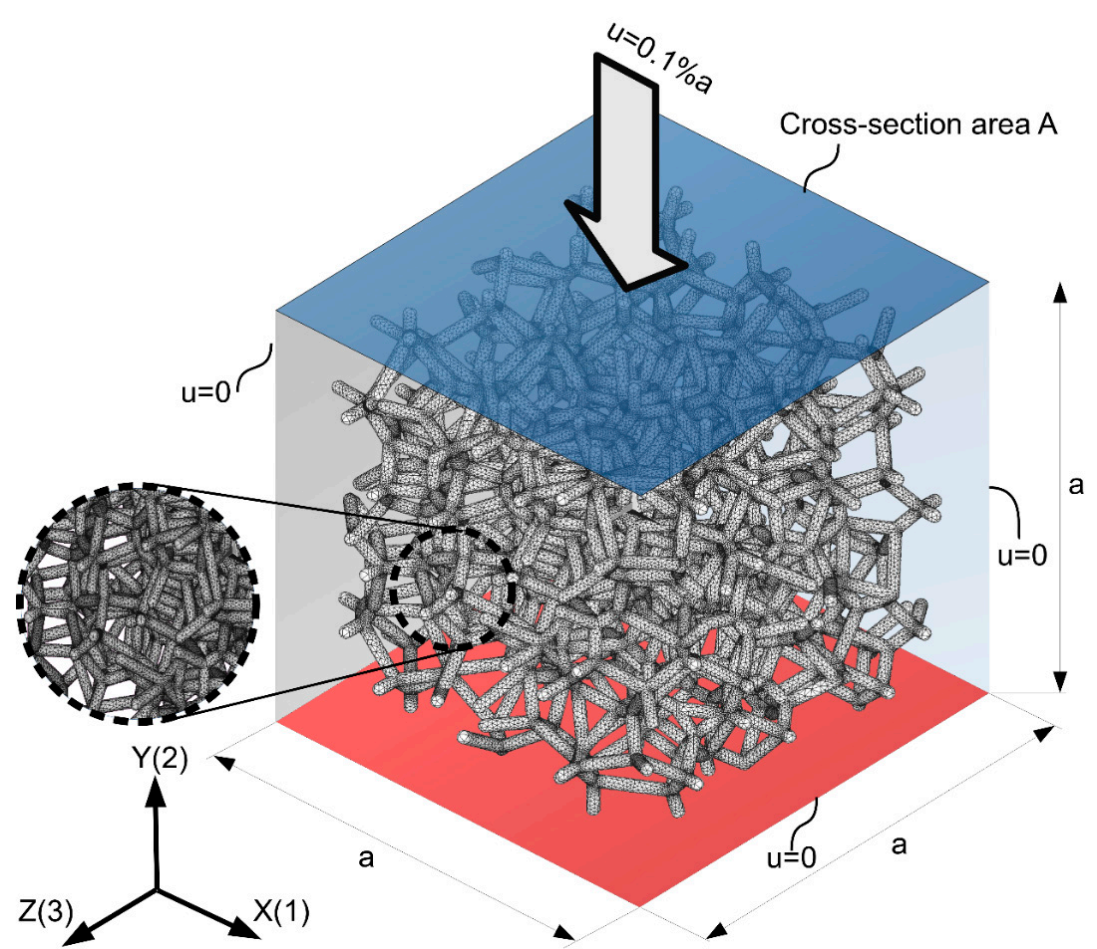
Illustration of representative volume element (RVE) and boundary conditions defined in FE simulations of uniaxial compression of open-cell metallic foams (porosity and CV(V) of the presented structure are 91.6% and 0.6, respectively).

The elastic properties of the analyzed metallic foams were simulated using the finite element method (FEM) in ANSYS software. For the skeleton of the foam material, the isotropic, elastic properties of pure aluminum were defined—Young’s modulus of *E* = 70 GPa and Poisson’s ratio *ν* = 0.33, cf. [21,42,43,44]. An example of a meshed model with a coordinate system and dimensions of the bounding box is depicted in Figure 3.

Boundary conditions corresponding to the uniaxial compression test along three perpendicular directions (1, 2, 3 coincident with the *X*, *Y*, and *Z* axis of the global coordinate system, respectively) were applied. Each of the LVT cellular structures was loaded with displacement equal to 0.1% of initial edge length *a* at one side and constrained on the opposite side with zero displacement in the actual direction of loading. Additionally, normal displacements of external nodes located close to each lateral face of the bounding box were coupled (see Figure 3). An algorithm for conducting the analyses was developed and implemented by means of the APDL language.

Geometrical and numerical contributions are closely dependent, and the goal was to distinguish an optimal mesh quality to obtain accurate results and not extend computational time. In the presented computations, geometrical and numerical convergence calculated according to [21] was obtained with finite element meshes of above 2 million elements. The estimated number of elements per structure was high enough and enabled us to use the linear type of finite elements.

The values of the macroscopic engineering stress have been evaluated as a quotient of the sum of the nodal reaction forces ΣF_n_ divided by the initial engineering cross section area A = a^2^. The engineering strain values were obtained by dividing the applied displacement by the initial height of the analyzed structure. Therefore, according to Hooke’s law, effective Young’s modulus E_eff_ was calculated as (2):

(2) $$E_{eff}=\frac{\sigma_{eng}}{\varepsilon }=\frac{\frac{\sum F_{n}}{A}}{\frac{u_{n}}{a}}=\frac{\frac{\sum F_{n}}{a^{2}}}{\frac{u_{n}}{a}}=\frac{\sum F_{n}}{au_{n}}$$

where σ_eng_ is engineering stress equal to the sum of reaction forces ΣF_n_ divided by an area of full cross section A, and ε is deformation calculated as displacement in normal direction un divided by initial edge length a.

## 3. Results and Discussion

### 3.1. Effective Young’s Modulus of Aluminum Open-Cell Foams

**Figure 4:**
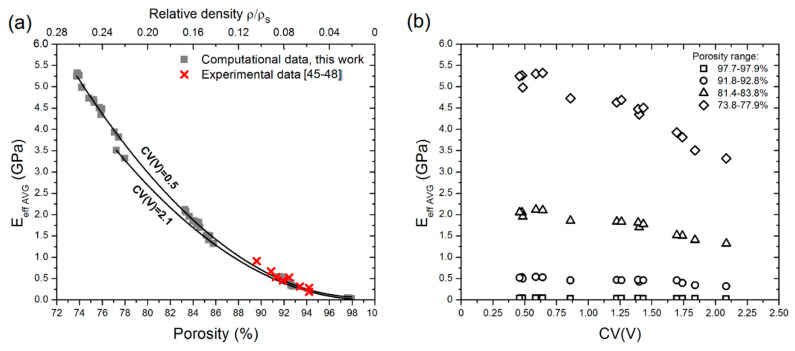
Average values of effective Young’s modulus E_eff AVG_ computed for designed aluminum open-cell foams versus (**a**) porosity and relative density and (**b**) coefficient of pore volume variation CV(V). Experimental values of E_eff AVG_ available for Duocel^®^ foams are indicated using ‘**x**’ symbols.

Values of effective Young’s modulus of designed aluminum open-cell foams, calculated for each of three orthogonal directions of compression, differed less than 10% from average ones. Therefore, anisotropy of effective Young’s modulus was not considered. The LVT models of open-cell foams with varied pore size distributions were designed since we found them the most reliable in mimicking the structure of ‘real’ foams. The randomly distributed pores with different sizes being close to an equiaxial shape enable obtaining the isotropy of the mechanical properties. Average values, hereinafter denoted as E_eff AVG_, were depicted versus porosity and pore volume variation coefficient (CV(V)) in Figure 4a,b, respectively.

Figure 4a shows a typical relationship between Young’s modulus and porosity (relative density) of open-cell structure, but in addition to different porosity, changes of E_eff AVG_ as a function of CV(V) depicted in Figure 4b are crucial. We also added experimental data points reported in the literature for Duocel^®^ commercial aluminum open-cell foams [45,46,47,48]. The structure of Duocel^®^ foams can be represented by LVT models in numerical simulations. Our computational prediction stays in good agreement with experimentally evaluated elastic modulus, although available only for a narrow range of porosity.

**Figure 5:**
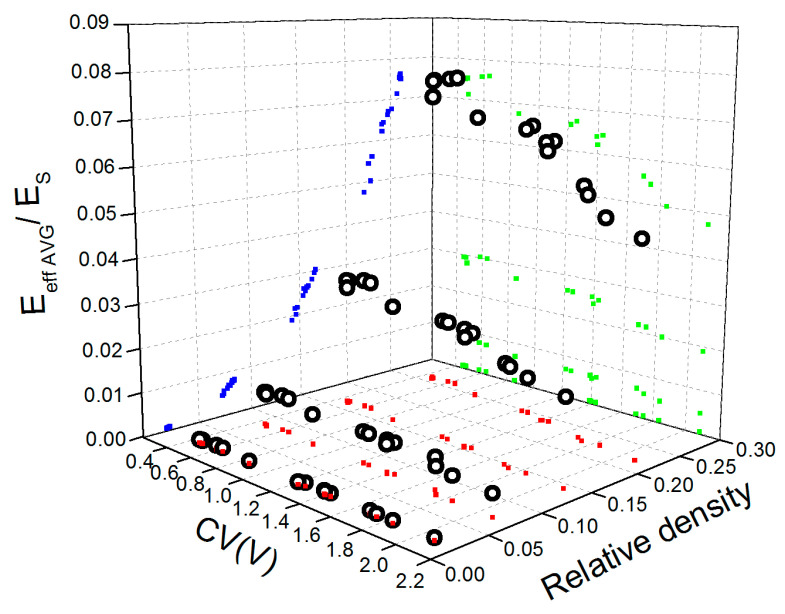
Average values of reduced Young’s modulus, E_eff AVG_/E_s_ (○), versus relative density and CV(V) calculated for the LVT aluminum open-cell structures and their xy (▪), yz (▪), and zx (▪) projections.

Over small ranges of porosity, decrement of E_eff AVG_ with increasing CV(V) was reported. More significant decrement is observed for lower porosity of approx. 74 to 84%, which corresponds to the ρ/ρ_S_ of 0.26 to 0.16, respectively. In light of this fact, the question arises of how to quantify the influence of structural nonuniformity on the elastic behavior of open-cell metallic foams. The general constitutive model proposed by Gibson–Ashby (Equation (1)) was considered because it has been derived to represent a relationship between reduced Young’s modulus and the relative density of porous materials. Fitting parameters C and n in the G–A model are considered structure dependent; thus, the influence of CV(V) on constants’ values was discussed in this paper. To this end, computed values of E_eff AVG_ were reduced by E_s_ and plotted versus both relative density ρ/ρ_S_ and CV(V) to show that Young’s modulus is not only dependent on the relative density but pore volume variation as well (see Figure 5).

**Figure 6:**
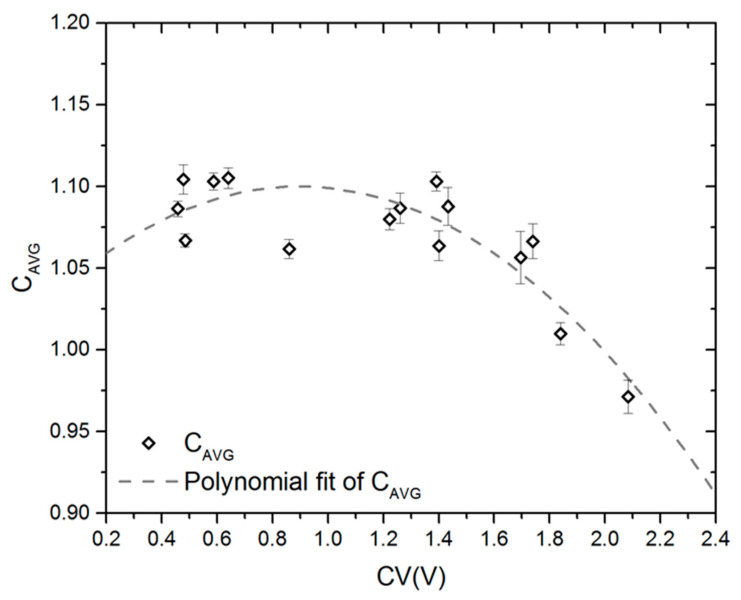
Average values of the scaling constant C_AVG_ of the Gibson–Ashby model computed for the LVT aluminum open-cell structures as a function of CV(V).

The value of the exponent n is broadly assumed as 2 for open-cell structures in many experimental reports available in the literature [34,35]. Nevertheless, the exponent is also a consequence of the deformation mechanism. When the struts are not aligned in the foam structure, bending at their intersection points (strut joints) or bending of the struts becomes dominant [33,34,35], and quadratic dependence of Young’s modulus on the relative density occurs. Computational data points reveal excellent fitting to the Gibson–Ashby model with the approximate value of R^2^ = 0.99 for each series. A coefficient corresponding to scaling factor C in the Gibson–Ashby model was calculated for each fitting curve y = ax^b^ (b = 2). Average values, denoted as C_AVG_, were plotted versus the coefficient of pore volume variation (CV(V)) in Figure 6.

Generally, the literature reports C = 1 and n = 2 for uniaxial compression tests of the elastic properties of open-cell foams. However, this set of fitting parameters was derived theoretically on the basis of the assumption of deformation only by bending cell edges. Moreover, the deformation was investigated on the basis of a geometrically simple, cubic model which does not resemble the structure of real foams properly but only represents them quantitatively. Hence, approaches incorporating more complex architectures have led to reporting values of scaling factor C even over the range of 2.1–2.2 [49] and 2.0–2.2 [50], or approx. 2.07 [51] for Kelvin cell and 2.3–2.4 [50] for Weaire–Phelan cell with nonuniform cell edge cross section. Buffel et al. in [50] also reported values of the C constant in the range 1.1–1.3 obtained as a result of calculations using the Kelvin cell model with uniform, circular cross sections of cell edges which correspond to the LVT architecture presented herein. Analysis of data points computed within this work allows us to state that the C constant may be correlated with the coefficient of pore volume variation (CV(V)) with the third-order polynomial fit. Numerical investigation of the influence of CV(V) reveals that values of C may be calculated from Equation (3).

(3) $$C=C_{0}+a_{1}\mathrm{CV}\left(V\right)+a_{2}\mathrm{CV}{\left(V\right)}^{2}$$

The fitting parameters C_0_ = 1.03, a_1_ = 0.15, and a_2_ = −0.08 were found to yield the best fit of the calculated data.

### 3.2. Effective Poisson’s Ratio of Aluminum Open-Cell Foams

**Figure 7:**
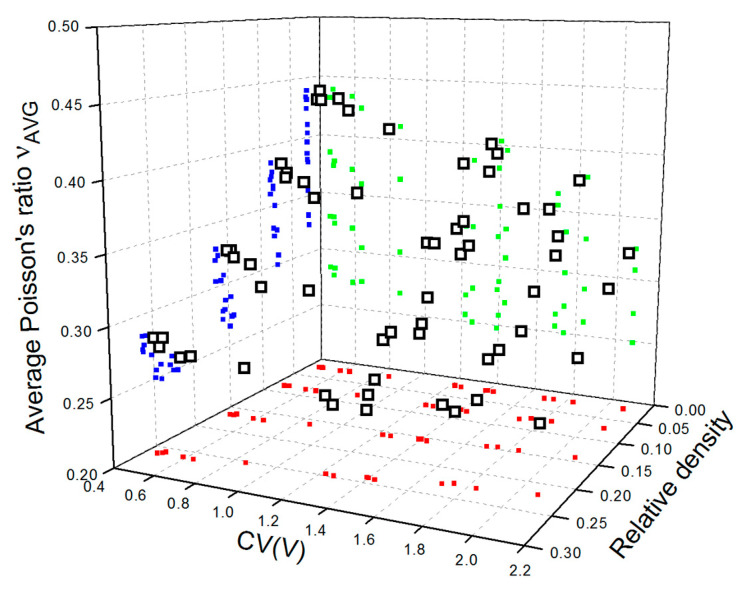
Average values of Poisson’s ratio ν_AVG_ (□) versus relative density and CV(V) calculated for designed aluminum open-cell foams and their xy (▪), yz (▪), and zx (▪) projections.

Data points representing values of average Poisson’s ratio calculated for the analyzed set of the LVT structures versus relative density and CV(V) are shown in Figure 7.

As it has been formerly reported [35,50], Poisson’s ratio of metallic open-cell foams is slightly dependent on relative density in such a manner that the relative density increases as Poisson’s ratio decreases. This is possible since, at low relative densities (low fraction of solid), the structure tends to be nearly incompressible and expand transversely to the direction of compression because of the bending of cell edges (struts). Calculated values of Poisson’s ratio also reveal a correlation with the pore size variation, similarly to effective Young’s modulus. In contrast to effective Young’s modulus, the influence of CV(V) becomes more evident at lower relative densities. Analysis of the graph in Figure 7 also allows us to state that the impact of relative density on Poisson’s ratio is weaker for metallic open-cell structures with greater variation of pore size.

## 4. Conclusions

In the present paper, structural nonuniformity quantified with the coefficient of pore volume variation (CV(V)) was investigated as a parameter affecting the elastic properties of metallic open-cell foams. Although the Laguerre–Voronoi tessellations resemble the structure of real foams, obtained values of effective Young’s modulus as the function of relative density represent perfect fitting to the Gibson–Ashby model, derived on simplified cubic model with cell edges subjected to bending during deformation. It was proven that the variation in size and spatial distribution of the pores is crucial for shaping the mechanical behavior of open-porous materials. Knowledge of the relationship between the fitting parameters C (given in the text) and n of the Gibson–Ashby model and microstructure is particularly important to be understood. The authors suggested the relationship between C and the coefficient of pore volume variation (CV(V)) for effective Young’s modulus estimation. According to presented computations, values of Eeff increase with increasing relative density but decrease with increasing CV(V). Thus, it was proven that the effective Young’s modulus is a function of both relative density ρ/ρS and structural inhomogeneity quantified with CV(V). Although Poisson’s ratio also depends on relative density ρ/ρS and CV(V), the relationship with relative density is reversed.

The presented approach can help to improve predictions of the elastic properties of metallic open-cell foams with respect to their structural inhomogeneity. It is a significant factor in both the design and optimization process of open porosity metallic structures for specific applications.
